# Sex-Change Chemicals and their Influence on the Brain

**DOI:** 10.1100/tsw.2001.362

**Published:** 2001-11-16

**Authors:** Catherine A. Harris

**Affiliations:** Brunel University, UK

**Keywords:** nonylphenol, gonadotropins, intersex, fish, endocrine disruption, xenoestrogen

## Abstract

The potential for man-made chemicals to mimic or antagonise natural hormones is a controversial issue, but one for which increasing amounts of evidence are being gathered worldwide. The controversy surrounds not so much the matter of whether these chemicals can mimic hormones in *vitro* — this phenomenon has been widely accepted in the scientific world — but more whether, as a result, they can disrupt reproduction in a wildlife situation. It has, nevertheless, been acknowledged that many wildlife populations are exhibiting reproductive and/or developmental abnormalities such as intersex gonads in wild roach populations in the U.K.[1] and various reproductive disorders in alligators in Lake Apopka, Florida[2]. However, the causative agents for many of these effects are difficult to specify, due to the extensive mixtures of chemicals — each of which may act via different pathways — to which wild populations are exposed, together with the wide variability observed even in natural (uncontaminated) habitats. As a result, any information detailing fundamental mechanism of action of the so-called endocrine disrupting chemicals (EDCs) is of use in determining whether or not these chemicals, as they are present in the environment, may in fact be capable of causing some of the effects observed in wildlife over recent years.

In this vein, a paper was recently published in *Environmental Science and Technology* providing evidence that an acknowledged (and widespread) xenoestrogen — 4-nonylphenol (NP) — can indeed affect reproductive parameters in the brain of fish[3]. Very few data are available showing that environmental oestrogens are capable of affecting the higher levels of the reproductive axis. However, this is an area that requires definition if we are to understand the actions of these chemicals in the reproductive growth and development of animals. The main route of NP into rivers is via sewage effluent, together with some industrial effluents, and it can be present in affected rivers at up to 180 µg/L[4] but is more commonly found at less than 10 µg/L.

One of the most widely accepted biomarkers of oestrogen exposure in fish is the synthesis of vitellogenin, a yolk precursor which is normally only found in maturing/adult females, but can be induced in males and juvenile females in response to an estrogenic stimulus. This response has been successfully exploited to determine the estrogenic nature of sewage effluents (e.g., Harries et al.[5]), but the implications of elevated vitellogenin production remain unclear. Some of the strongest evidence indicating that the reproductive health of wild fish in the U.K. may be at risk from exposure to EDCs in effluents is shown in studies undertaken by Jobling and colleagues[1]. These data demonstrate a link between the concentration of effluent in rivers and the proportion of intersex (defined by the simultaneous presence in the gonad of both male and female tissue types) roach sampled therein. The situation was particularly striking in the River Aire in Yorkshire — notorious with respect to NP contamination — where 100% of 'male' roach displayed some extent of intersex condition. Currently, it is unclear as to at which life-stage this disruption of gonadal development occurred; could it be a continuous process throughout the life of the fish or is there a critical window in the early stages of development? This question is being addressed, as is that of the unknown reproductive capabilities of such intersex fish.

To date, most studies have reported effects of environmental oestrogens occurring in the target organ (e.g., intersexuality in the gonads, or vitellogenesis in the liver), but oestrogen receptors are also present in the brain (hypothalamus) and pituitary gland, and are important for controlling the synthesis and secretion of the reproductive hormones follicle-stimulating hormone (FSH) and luteinising hormone (LH); these hormones essentially control gonadal growth and maturation, respectively. The recent study by Harris et al.[3] found that fish exposed to environmentally realistic concentrations of NP (0.7, 8.3 and 85.6 µg/L) had suppressed plasma FSH levels. Since FSH in fish is thought to be controlled by a negative feedback response to steroid hormones, it seems likely that the NP was capable of penetrating the brain, such that it behaved as a natural oestrogen would in suppressing the levels of this hormone. In addition, the fish exposed to the highest concentration of NP over the 18-week period — during which their ovaries would normally start to mature — had markedly underdeveloped ovaries. In fact, their ovaries had not grown at all since the start of the experiment, whereas those of the fish in the control tank had. This may have been primarily a result of the suppression of FSH in the exposed group, since FSH is thought to mediate oocyte recruitment[6] and also stimulate uptake of vitellogenin into developing oocytes[7].

So, where do the studies indicating an effect of NP on gonadotropins fit in with the general picture on intersex fish? The role of gonadotropins in gonadal development of fish is not yet wholly understood, due to the relatively recent discovery of 'developmental' gonadotropin, FSH, in fish[8,9], and also the limitations of the currently available assays. However, FSH producing cells have been found to appear early in development of rainbow trout[10], prior to the first signs of gonadal sex differentiation in these fish. It is therefore possible that FSH plays a part in early gonadal development in rainbow trout. In juvenile fish, the role of gonadotropins in puberty has likewise not yet been defined, since in most cases they have not been detected in immature fish (due to the limitations of the available assays). In humans, however, gonadotropins become significant at puberty, when they induce the secretion of hormones from the gonads which in turn initiate the maturation of the reproductive system and development of the secondary sex characteristics. If a similar scenario were to ensue in teleosts, the implications of inhibition of gonadotropin secretion following exposure to xenoestrogens for reproductive development would be profound.
